# Anaemia and depression before and after birth: a cohort study based on linked population data

**DOI:** 10.1186/s12888-018-1796-6

**Published:** 2018-07-13

**Authors:** Fenglian Xu, Lynette Roberts, Colin Binns, Elizabeth Sullivan, Caroline S. E. Homer

**Affiliations:** 1 Faculty of Health, University of Technology Sydney, Ultimo, 2007 Australia; 2 Discipline of Clinical Psychology, Graduate School of Health, University of Technology Sydney, Ultimo, 2007 Australia; 3 School of Public Health, Curtin University, Perth, Australia

**Keywords:** Anaemia, Depression, Risk factors, Women, Birth

## Abstract

**Background:**

To investigate the rates of hospitalisation for anaemia and depression in women in the six-year period (3 years before and after birth). To compare hospital admissions for depression in women with and without anaemia.

**Methods:**

This is a population-based cohort study. Women’s birth records (New South Wales (NSW) Perinatal Data Collection) were linked with NSW Admitted Patients Data Collection records between 1 January 2001 and 31 December 2010, so that hospital admissions for mothers could be traced back for 3 years before birth and followed up 3 years after birth. Setting: NSW Australia. Subjects: all women who gave birth to their first child in NSW between 1 January 2004 and 31 December 2008.

**Results:**

Hospital admissions for both anaemia and depression were increased significantly in the year just before and after birth compared with the years before and after. Women with anaemia were more likely to be admitted to hospital for depression than those without (for principal diagnosis of depression, adjusted OR = 1.62, 95% CI = 1.25–2.11; for all diagnosis of depression, adjusted OR = 2.01, 95% CI = 1.70–2.38).

**Conclusions:**

Depression was associated with anaemia in women before and after birth. This finding highlight the important role of primary care providers in assessing for both anaemia and depressive symptomatology together, given the relationship between the two. Treating or preventing anaemia may help to prevent postnatal depression.

## Background

Depression in the postnatal period is a major health problem [1–3], with adverse outcomes for the mother and baby [4] including bonding [5–8], child’s later socioemotional development [9] academic achievement [10] and risk for psychopathologies [9]. One in every seven or eight women (around 12–16%) will experience depressive symptoms in the post-partum period [11–14], costing the Australian economy $113.86 million annually [13]. We have previously shown that the hospital admission rates for psychiatric disorders increased in the first year after birth significantly between 2001 and 2010 (1.16% in 2001, 2.28% in 2010) [15] in New South Wales (NSW) of Australia. The hospital admission increase was mainly attributed to unipolar depression, adjustment disorders and anxiety disorders [15].

Poor physical health is a risk factor for postnatal depression (PND). A prospective cohort study in1,507 nulliparous women in Melbourne of Australia showed that women with five or more physical health problems had a six-fold increase in the risk of depressive symptoms at 3 months postpartum and a three-fold increase in the risk of subsequent depressive symptoms at 6–12 months postpartum [14]. Women experiencing a greater number of physical health problems may be at an increased risk of postnatal depression.

One of these physical health risk factors is anaemia, a common problem for women (global prevalence of 29%) [16], particularly in pregnancy (global prevalence of 38%) [16] due to additional physiological demands on the mother. A population-based cross-sectional study of all singleton births (*n* = 511,938) in Finland between 2002 and 2010 reported that anaemia is a predictor of depression during pregnancy [17]. A prospective cohort study in Spain (*n* = 729) found that mothers with low levels of the iron marker, ferritin, at 48 h post-partum were 3.73 times more likely to have PND at 32 weeks [18]. Another prospective study in American women found that low concentrations of hemoglobin in mothers at 7 days postpartum correlated with depressive symptoms at 28 days postpartum [19]. However, a study in Chinese mothers found no association between iron levels in pregnancy and 3 days postpartum, with depression scores 24–48 h or 6 weeks after delivery [20].

Given the wide-reaching impact and costs on the individual, parent-child dyad and community, we sought to investigate whether there is an association between anaemia and depression in Australia. These findings could inform public health policy, including highlighting a potential link between preventable physical and mental health problems in the perinatal period. Specifically, we used population-based linked data in Australia to:

1. Describe the rates of hospitalization for anaemia and depression in mothers in the 3 years before and after giving birth.
2. Compare the association of hospital admission of depression in women with and without anaemia.

We hypothesized that anaemia is associated with an increase in hospital admissions for depression.

## Methods

### Study population and design

**Fig. 1:**
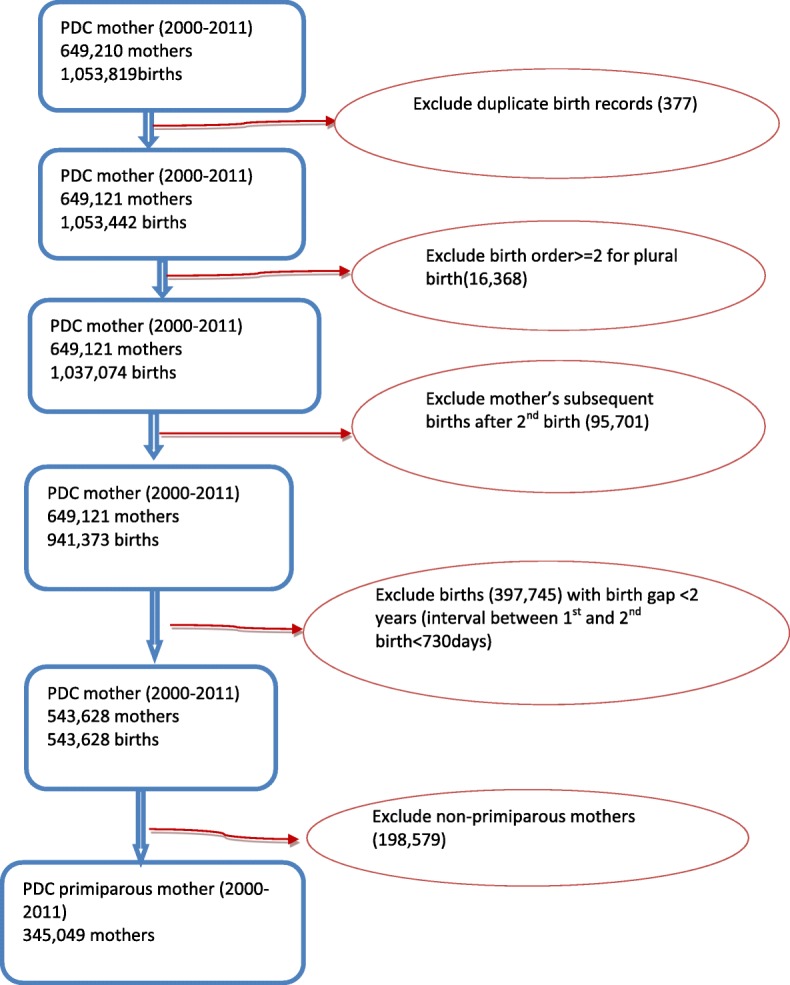
The study population selection

**Fig. 2:**
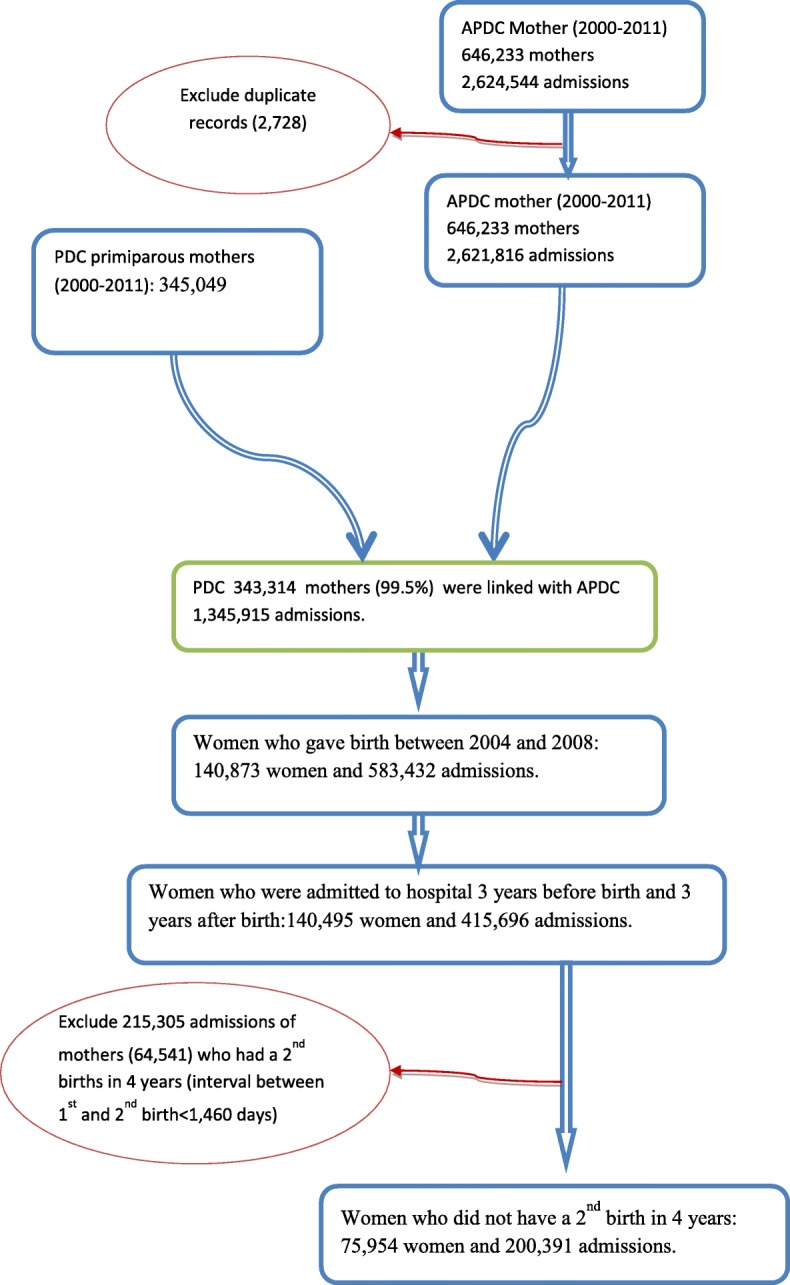
The data linkage and study population

This is a population-based cohort study, including all women who gave birth to their first child in New South Wales (NSW) between 1 January 2004 and 31 December 2008. Mothers who had subsequent births in 4 years after the first birth were excluded. Data were linked using the NSW Perinatal Data Collection (PDC) and the NSW Admitted Patients Data Collection (APDC). Women’s hospital admissions for anaemia and depressive disorders were identified by APDC records. Mother’s birth records were linked with APDC records between 1 January 2001 and 31 December 2010, so that hospital admissions for mothers could be traced back for 3 years before birth and followed up 3 years after birth. The study population and data linkage are detailed in Figs. 1 and 2.

The PDC is a data collection of births of at least 20 weeks gestation or at least 400 g birthweight in NSW of Australia. It includes all births in hospitals (public and private) and home births. The database includes information on maternal characteristics and outcomes of pregnancy, labour and birth. The APDC is a routinely collected census of all hospital separations in NSW hospitals including public, private and psychiatric hospitals and day procedures. It covers all patient hospitalisations and includes information on patient demographics, diagnoses and clinical procedures. The diagnoses for admissions have been coded according to the 10th revision of the International Statistical Classification of Diseases and Related Health Problems, Australian Modification (ICD-10-AM) [21].

Data linkage was carried out by the NSW Department of Health Centre for Health Record Linkage (CHeReL). Each record from PDC and APDC datasets was assigned a Person Project Number to allow records for the same individual to be linked. In the 1000 randomly selected sample of records, the false positive rate of the linkage was 0.3% and false negative < 0.5%.

### Diagnosis

The diagnoses in each hospital admission record in APDC data include 55 diagnoses (one principal diagnosis, one stay diagnosis and 53 other diagnoses). Principal diagnosis refers to the one which was chiefly responsible for hospital admissions [22]. The stay diagnosis refers to the one that most influenced the length of stay in hospital [15]. Other diagnosis is an additional diagnosis. It refers to a condition or a complaint coexisting with the principal and stay diagnoses or arising during the hospitalisation [15]. Hospital admission refers to hospitalization (a patient was admitted to hospital for inpatient service). PND refers to the principal diagnoses of depression after birth. Anaemia refers to all diagnoses of anaemia (including principal and non-principal diagnoses). Mothers with anaemia were identified using ICD-10-AM diagnosis codes: (1) D50-D53 [nutritional anaemia]; (2) D55-D59 [haemolytic anaemia], (3) D60-D64 [aplastic and other anaemia] and O99.0 [anaemia complicating pregnancy, childbirth and the puerperium]. Mothers with depressive disorders were identified using ICD-10-AM diagnosis codes: (1) F32-F33 [depressive disorder] and (2) F53 [Mental and behavioural disorders associated with the puerperium]. Only the first hospital admission with the diagnosis of *anaemia* or depressive disorders was used for the analysis of the rate, odds ratio (OR) and adjusted OR.

### Statistics

Descriptive statistics were used to summarise the rate and proportion of hospital admissions. The proportion was calculated by women’s 1st hospital admissions for the diagnosis of anaemia or depression as nominator and overall hospital admissions as denominator. Logistic regression models were used to estimate the odds ratio (OR) of anaemia on hospitalisation for depression. The dependent variable is the first hospital admission for depression (yes/no) in the study period (3 years before birth and 3 years after the birth). The independent variable is the first hospital admission for all diagnoses of anaemia in the study period. The adjusted factors for the OR include maternal age, maternal country of birth, maternal diabetes mellitus and hypertension, gestational diabetes, smoking status during pregnancy, remoteness of living area,, method of birth, gestational age, baby’s death and place of birth and the Index of Relative Socio-economic Disadvantage Quintile [23]. The analyses were conducted using IBM SPSS (Statistical Package for Social Science) Statistics 20.

## Results

Characteristic of the study population and the rate of the first hospital admissions for anaemia are detailed in Table 1. Young women (< 20 years old) were more likely to be admitted to hospital with the diagnosis of anaemia than those aged between 25 and 39 years old. The mothers in disadvantaged group; with maternal diabetes mellitus or hypertension were also more likely to be admitted to hospitals with the diagnosis of anaemia. Mothers with anaemia were more likely to have stillbirth, preterm birth and their babies were more likely to die in neonatal/postnatal period.

**Table 1:** Primiparous mother’s characteristic and the 1st hospital admissions for anaemia 2001–2010, Australia

Factor	Value	Mother	Admission for anaemia	Rate (%)	95% CI	
Maternal age*	< 20	6284	262	4.17	3.68	4.66
	20–24	14,350	521	3.63	3.32	3.94
	25–29	19,833	662	3.34	3.09	3.59
	30–34	20,472	644	3.15	2.91	3.39
	35–39	11,479	364	3.17	2.85	3.49
	40+	3529	116	3.29	2.70	3.88
	Total	75,947	2569	3.38	3.25	3.51
Women’s country of birth	Australia	48,331	1589	3.29	3.13	3.45
	Other countries	27,623	980	3.55	3.33	3.77
	Total	75,954	2569	3.38	3.25	3.51
Remoteness*	Major cities	53,399	1827	3.42	3.27	3.57
	Inner regional	16,092	497	3.09	2.82	3.36
	Out regional and remote	5252	202	3.85	3.33	4.37
	Total	74,743	2526	3.38	3.25	3.51
Smoking during pregnancy	No	65,899	2224	3.37	3.23	3.51
	Yes	9694	327	3.37	3.01	3.73
	Total	75,593	2551	3.37	3.24	3.50
Index of Relative SE Disadvantage Quintile*	1st quintile least disadvantaged	17,196	487	2.83	2.58	3.08
	2.00	15,490	501	3.23	2.95	3.51
	3.00	14,437	516	3.57	3.27	3.87
	4.00	12,953	455	3.51	3.19	3.83
	5th quintile most disadvantaged	14,667	567	3.87	3.56	4.18
	Total	74,743	2526	3.38	3.25	3.51
Maternal diabetes mellitus*	No	75,488	2543	3.37	3.24	3.50
	Yes	466	26	5.58	3.50	7.66
	Total	75,954	2569	3.38	3.25	3.51
Gestational diabetes	No	72,051	2417	3.35	3.22	3.48
	Yes	3903	152	3.89	3.28	4.50
	Total	75,954	2569	3.38	3.25	3.51
Maternal hypertension*	No	75,175	2529	3.36	3.23	3.49
	Yes	779	40	5.13	3.58	6.68
	Total	75,954	2569	3.38	3.25	3.51
Delivery mode	Vaginal	49,827	1641	3.29	3.13	3.45
	Caesarean section	26,073	928	3.56	3.34	3.78
	Total	75,900	2569	3.38	3.25	3.51
Baby’s death*	None	75,401	2526	3.35	3.22	3.48
	Still birth	340	23	6.76	4.09	9.43
	Neonatal/post neonatal death	151	16	10.60	5.69	15.51
	Total	75,892	2565	3.38	3.25	3.51
Gestational age (week)*	< 28	534	39	7.30	5.09	9.51
	28–31	696	50	7.18	5.26	9.10
	32–36	5153	221	4.29	3.74	4.84
	37–41	68,023	2189	3.22	3.09	3.35
	42w+	1540	70	4.55	3.51	5.59
	Total	75,946	2569	3.38	3.25	3.51
Place of birth*	Hospital	74,318	2512	3.38	3.25	3.51
	Birth Centre	1482	46	3.10	2.22	3.98
	Others	145	11	7.59	3.28	11.90
	Total	75,945	2569	3.38	3.25	3.51

*SE* Socioeconomic

*Significantly associated with the 1st hospital admissions with anaemia by chi square test (*p* < 0.05)

There were 236 hospital admissions with principal diagnosis of anaemia and 2569 admissions with all diagnosis of anaemia in the study periods (3 years before birth and 3 years after birth) (Table 2). The admissions with principal diagnosis of anaemia only accounted for 9.19% (236/2569) of the hospital admission for all anaemia diagnoses. That is, more than 90% anaemia was diagnosed as a non-principal diagnoses.

**Table 2:** The rate of women’s 1st hospital admissions with the diagnosis of anaemia before and after birth (*n* = 75,954) 2001–2010, Australia

Period		Principal diagnosis	Rate (/1000)	95% CI		All diagnosis	Rate (/1000)	95% CI	
Before birth	3rd year	25	0.33	0.2	0.46	77	1.01	0.78	1.24
	2nd year	21	0.28	0.16	0.4	88	1.16	0.92	1.4
	1st year	63	0.83	0.63	1.03	1220	16.06	15.17	16.95
After birth	1st year	71	0.93	0.71	1.15	999	13.15	12.34	13.96
	2nd year	23	0.30	0.18	0.42	88	1.16	0.92	1.4
	3rd year	33	0.43	0.28	0.58	97	1.28	1.03	1.53
Total		236	3.11	2.71	3.51	2569	33.82	32.53	35.11

**Fig. 3:**
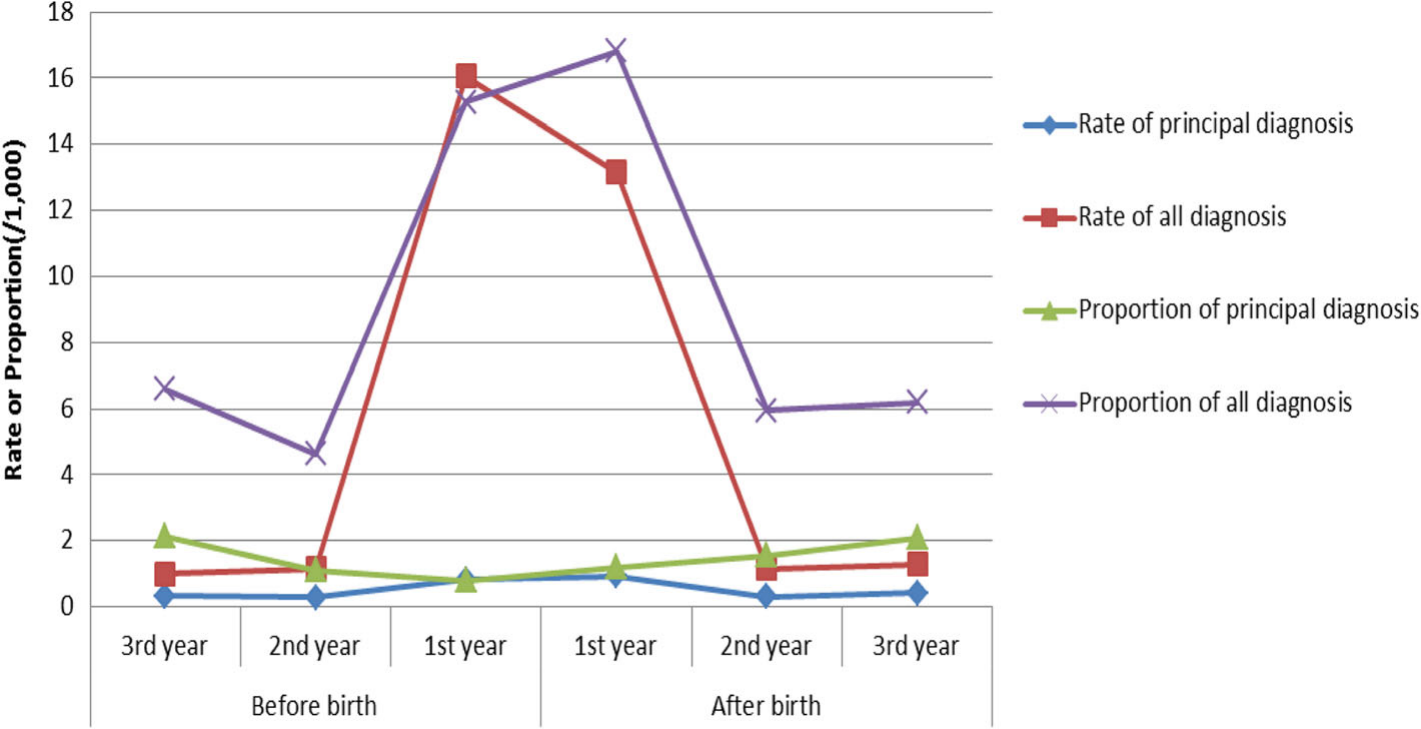
The rate and proportion of women’s hospital admissions for anaemia 2001–2010, Australia

The rates of anaemia in women in the year just before and after giving birth were significantly higher than the 2 years before (the second and third year before birth) and 2 years after (the second and third year after birth) the period, especially for all diagnoses of anaemia (Table 2 and Fig. 3).The hospital admission rate for the principal diagnosis of anaemia was 0.83 per 1000 women (95% CI = 0.63–1.03/1000) in the year just before birth; 0.93 per 1000 women (95% CI = 0.71–1.15/1000) in the year just after birth. The hospital admission rate for all diagnosis of anaemia was 16.06 per 1000 women (95% CI = 15.17–16.95/1000) in the year just before the birth; 13.15 per 1000 women (95% CI = 12.34–13.96/1000) in the year just after birth (Table 2).

The proportion of women’s anaemia in the year just before and after birth were also significantly higher than the 2 years before (the second and third year before birth) and 2 years after (the second and third year after birth) this period for all diagnoses of anaemia (Table 3 and Fig. 3). For the principal diagnosis of anaemia, the proportion in the year just before the birth (0.79/1000 admission, 95% CI = 0.60–0.98) was lower than the third year before birth (2.14/1000 admission, 95% CI = 1.30–2.98) and the third year after birth(2.10/1000 admission, 95% CI = 1.38–2.82); The proportion in the year just before the birth was not significantly different from the second year before birth and the first 2 years after birth.

**Table 3:** The proportion of women’s 1st hospital admissions with the diagnosis of anaemia in all hospital admissions 2001–2010, Australia

Period		Hospital admissions	Principal diagnosis	Proportion (/1000)	95% CI		All diagnosis	Proportion (/1000)	95% CI	
Before birth	3rd year	11,671	25	2.14	1.30	2.98	77	6.6	5.13	8.07
	2nd year	18,965	21	1.11	0.64	1.58	88	4.64	3.67	5.61
	1st year	79,903	63	0.79	0.60	0.98	1220	15.27	14.42	16.12
After birth	1st year	59,383	71	1.2	0.92	1.48	999	16.82	15.79	17.85
	2nd year	14,790	23	1.56	0.92	2.20	88	5.95	4.71	7.19
	3rd year	15,679	33	2.1	1.38	2.82	97	6.19	4.96	7.42
Total		200,391	236	1.18	1.03	1.33	2569	12.82	12.33	13.31

**Fig. 4:**
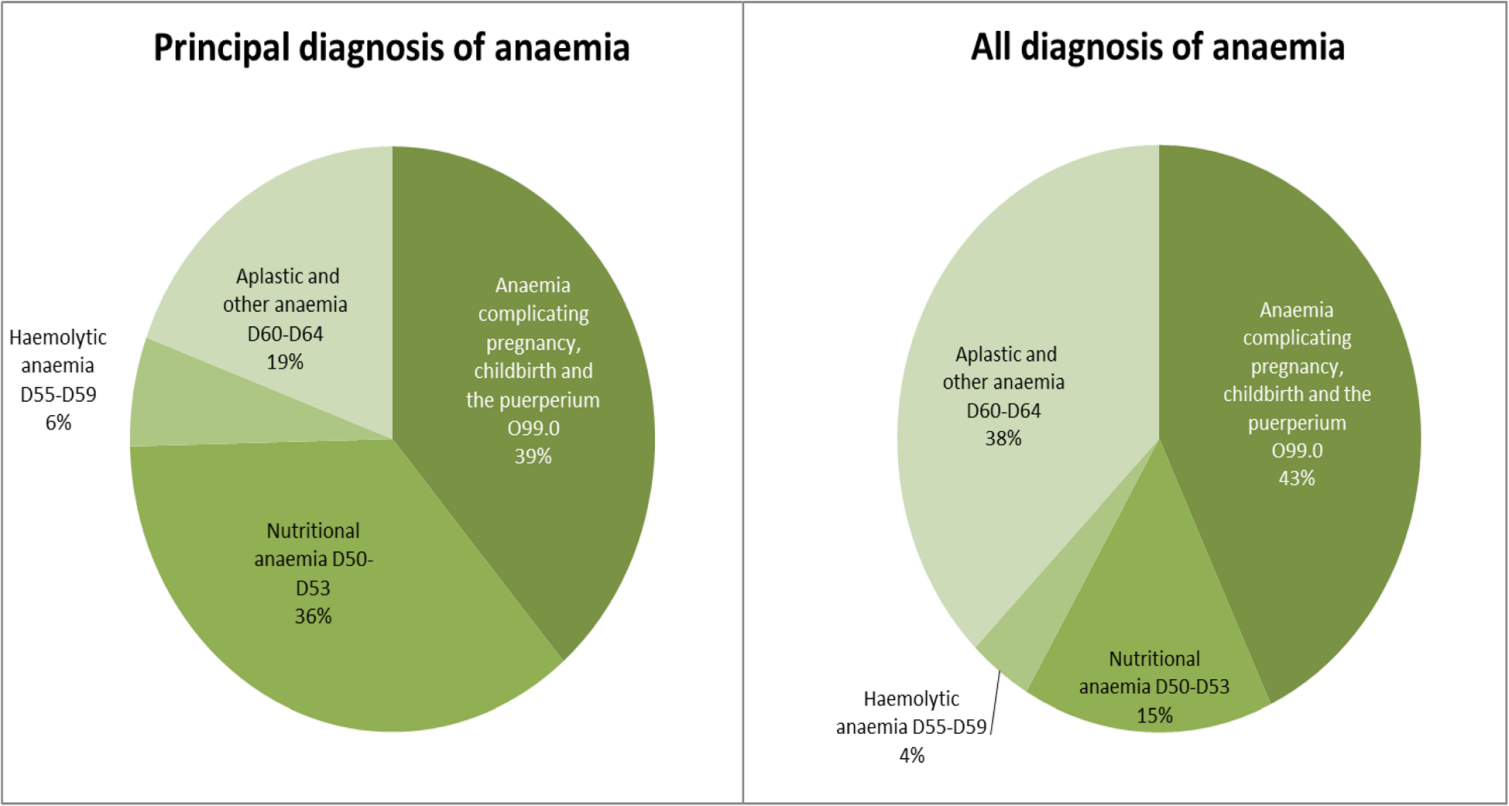
The percentage of diagnoses of anaemia for women’s hospital admissions before and after birth 2001–2010, Australia

Figure 4 shows that majority of the anaemia were attributed to anaemia complicating pregnancy, childbirth and the puerperium (38.71% for principal diagnosis and 42.99% for all diagnosis) and nutritional anaemia (35.89% for principal diagnosis and 15.47% for all diagnosis). Haemolytic anaemia accounted for 6.05% for principal diagnosis and 3.94% for all diagnosis of anaemia. Figure 4 also shows that there was more unspecified diagnosis of anaemia in non-principal diagnoses.

Table 4 shows the first hospital admissions for depression before and after birth. Compared with the hospital admissions before birth, the hospital admissions for depression increased significantly in the first and second year after birth. Women in the pregnancy year were less likely to be admitted to hospital compared with the 2 years before pregnancy and 3 years after birth. For principal diagnosis, the first hospital admission rate of depression was 8.94 per 1000 Person Year (PY) (95% CI = 8.27–9.61) in the first year after birth; while the rate of depression was only 0.91/1000PY (95% CI = 0.70–1.12) in the year before birth. For all diagnosis, the rate of depression was 16.02 per 1000 Person Year (PY) (95% CI = 15.12–16.92) in the first year after birth; while the rate of depression was only 6.48/1000PY (95% CI = 5.91–7.05) in the year before birth.

**Table 4:** The 1st Hospital admissions for depressive disorders in 3 years before and after birth 2001–2010, Australia

Period		Mothers (PY)	Principal diagnosis	Rate (/1000PY)	95% CI		Mothers (PY)	All diagnosis	Rate (/1000PY)	95% CI	
Before birth	3rd year	75,954	96	1.26	1.01	1.51	75,954	229	3.01	2.62	3.4
	2nd year	75,858	85	1.12	0.88	1.36	75,725	207	2.73	2.36	3.10
	1st year	75,773	69	0.91	0.70	1.12	75,518	489	6.48	5.91	7.05
After birth	1st year	75,704	677	8.94	8.27	9.61	75,029	1202	16.02	15.12	16.92
	2nd year	75,027	158	2.11	1.78	2.44	73,827	226	3.06	2.66	3.46
	3rd year	74,869	85	1.14	0.90	1.38	73,601	121	1.64	1.35	1.93
Total		453,185	1170	2.58	2.43	2.73	449,654	2474	5.50	5.28	5.72

*PY* Refers to person year, *CI* Refers to confidence interval

**Fig. 5:**
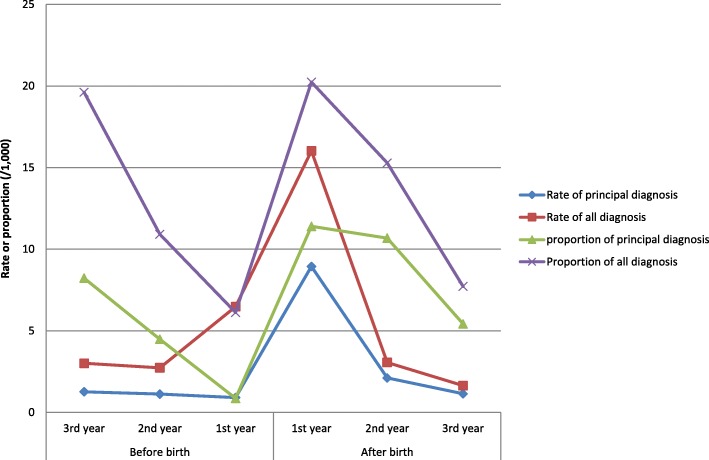
The rate and proportion of women’s hospital admissions for depression 2001–2010, Australia

To consider the impact of the increase of overall hospital admissions during pregnancy and perinatal period, the proportion of depression was analysed (Table 5). Similar to the change of rate in Table 4, the proportion of hospital admissions for depression increased significantly in the first and second year after birth. Women’s hospital admissions for depression in the year just before birth were the lowest compared with the 2 years before and 3 years after (Table 5 and Fig. 5).

**Table 5:** The proportion of women’s 1st hospital admissions with the diagnosis of depression in all hospital admissions 2001–2010, Australia

Period		Hospital admissions	Principal diagnosis	Rate (/1000PY)	95% CI		All diagnosis	Rate (/1000PY)	95% CI	
Before birth	3rd year	11,671	96	8.23	6.59	9.87	229	19.62	17.1	22.14
	2nd year	18,965	85	4.48	3.53	5.43	207	10.91	9.43	12.39
	1st year	79,903	69	0.86	0.66	1.06	489	6.12	5.58	6.66
After birth	1st year	59,383	677	11.40	10.55	12.25	1202	20.24	19.11	21.37
	2nd year	14,790	158	10.68	9.02	12.34	226	15.28	13.3	17.26
	3rd year	15,679	85	5.42	4.27	6.57	121	7.72	6.35	9.09
	Total	200,391	1170	5.84	5.51	6.17	2474	12.35	11.87	12.83

Figure 5 shows that the difference of the proportion and rate of depression for both principal and all diagnosis. The proportions of depression (including both principal and all diagnosis) decreased significantly in the first year before birth compared with the rates of depression.

Table 6 shows the association between anaemia and depression in women. Women with anaemia were more likely to be admitted to hospital for depression (for principal diagnosis of depression, adjusted OR = 1.62, 95% CI = 1.25–2.11) (for all diagnosis of depression, adjusted OR = 2.01, 95% CI = 1.70–2.38) than women without anaemia.

**Table 6:** The odds ratios (OR) of hospital admissions for depressive disorders in women with and without anaemia 2001–2010, Australia

Diagnoses of depression	Hospital admissions for anaemia	N	Hospital admissions for depression	Rate (%)	Crude OR	95% CI		Adjusted OR	95% CI	
Principal diagnosis	No	73,385	1109	1.51	1			1		
	Yes	2569	61	2.37	1.59	1.22	2.06	1.62	1.25	2.11
	Total	75,954	1170	1.54						
All diagnosis	No	73,385	2317	3.16	1			1		
	Yes	2569	157	6.11	2.00	1.69	2.36	2.01	1.70	2.38
	Total	75,954	2474	3.26						

The adjusted OR was adjusted for maternal age, maternal country of birth, maternal diabetes mellitus and hypertension, gestational diabetes, smoking status during pregnancy, remoteness of living area, delivery method, gestational age, baby’s death, place of birth and a socioeconomic indicator (i.e. the Index of Relative Socio-economic Disadvantage Quintile)

## Discussion

This study showed that the hospital admissions for depression increased significantly in the first year after birth compared with those before birth. The increased trend after birth is consistent with our previous study [15] and other studies from other areas [24]. For depression, the proportions (including both principal and all diagnosis) decreased significantly in the year before birth because of the increase of overall hospital admissions.

This study also showed that hospital admissions for anaemia with all diagnosis were significantly increased in the year of pregnancy (16.06 per 1000 women, 95% CI = 15.17–16.95/1000) and the first year after birth (13.15 per 1000 women, 95% CI = 12.34–13.96/1000). To consider the impact of increased overall hospital admissions before and after birth, the proportion of anaemia was also analyzed. Similar to the hospital admission rate, the proportion of anaemia was also significantly increased in the year of pregnancy and the first after birth. A systematic analysis of population-representative data from 107 countries between 1995 and 2011 showed that global anaemia prevalence was between 29% (95% credibility intervals (CI) = 24–35) and 33% (95% CI = 29–37) in non-pregnant women, 38% (95% CI = 34–43) and 43% (95% CI = 39–47) in pregnant women [16]. In high-income regions (including Australia), anaemia prevalence was between 14% (95% CI = 12–18) and 16% (95% CI = 12–22) in non-pregnant women, 22% (95% CI = 16–29) and 23% (95% CI = 18–30) in pregnant women [16]. The rate of anaemia in NSW women was significantly lower than other areas [16]. This study imply that majority of women with Anaemia were not admitted to hospitals.

Majority of anaemia (91%) was diagnosed as a non-principal diagnosis rather than principal diagnosis, as many women with anaemia were not diagnosed until they were admitted into hospital to give birth or for other medical reasons. Thus the greater the number of hospital admissions for other principal diagnoses, the greater the number of anaemia cases that will be found as an additional diagnosis. In New South Wales, 99.5% women gave birth in hospitals, this provided a good opportunity to identify women with anaemia. To adjust the impact of overall hospital admissions, the proportion of hospital admissions for anaemia (overall hospital admissions as denominator) was calculated.

Women in the pre- and postnatal periods are particularly vulnerable to the effects of iron deficiency on mood through both direct and indirect pathways [25]. Factors such as peripartum blood losses, postpartum haemorrhaging, increased nutritional demands to support fetal developmental and breastfeeding, may cause iron-deficiency [25, 26], which may disrupt the functioning of neurotransmitters which implicated in mood.

This study showed that women with anaemia were more likely to be admitted to hospital for depression than those without. The finding are consistent with the results based on population-based cohort studies from Finland [17] and Spain [18]. Correspondingly, we see an association between iron-deficiency and mood across a range of studies, such as high rates of anaemia in psychiatric patients with chronic depression [2], and conversely, higher rates of depression in people with anaemia [1, 27]. Lever-van Milligen and colleagues found that anaemia status was associated with depression [28].

Potential mechanisms of effect for the relationship between anaemia and depression may associated with the role of iron in mood regulation. Iron contributes to myelination in the brain, as well as the development and functioning of the monamine system, which are key neurotransmitters involved in mood such as serotonin, dopamine and noradrenaline [29, 30]. Iron deficiency alters the signalling, production and metabolism of these monamines, including other key excitatory and inhibitory neurotransmitters such as glutamate and γ-aminobutyric acid respectively, which govern brain activity [29]. Imbalances in the concentration of these neurotransmitters then affect emotion regulation leading to depressed mood and behaviour [31–33]. Research with iron-deficient mothers in Africa found that iron supplementation successfully treated mood and cognitive disturbances [34].

In addition to this proposed direct pathway, iron deficiency and depression may be associated via an indirect, stress-induced inflammatory pathway. The individuals with depression experience chronic psychosocial and physical stressors. The stress lead to an overactive hypothalamic–pituitary–adrenal (HPA) axis, which triggers stress-induced elevated levels of pro-inflammatory cytokines [35–38]. Pro-inflammatory cytokines interfere with the functioning of monamines, resulting in a reduction of available neurotrasmitters [39–42]. This inflammatory response also affects iron metabolism, with depressed patients showing reduced hemoglobin (Hb) levels and red blood cell count relative to controls [43, 44].

### Strengths and limitations

The strengths of this study include the population-based methodology, and data spanning a 6 year period to offer a richer understanding of mental and physical health in Australian women. Our findings present a baseline period from which to monitor the association between anaemia and depression in Australian women.

These findings also need to be considered within the context of study limitations.

This study shows that, for the principal diagnosis of anaemia, the proportion in the year just before the birth was lower than the third year before birth and the third year after birth. This was because women’s hospital admissions increased significantly in the pregnancy year (more than four times than the second and third year before birth and the second and third years after birth), however the absolute hospital admissions were still higher than the 2 years before and the 2 years after the year of birth. This study further showed that the proportion of depression for both principal and all diagnosis decreased significantly in the first year before birth (Fig. 5). This was because of the increase of women’s hospital admission in the year of pregnancy. The rates of depression for both principal and all diagnosis were less likely to be impacted by the number of overall admissions because the denominator for the rate analysis was the number of women rather than admissions. For anaemia, the proportion did not decrease as significantly as depression because the number of hospital admissions with the diagnoses of anaemia also increased significantly in the year of pregnancy (Fig. 3).

The ‘other anaemia’ diagnosis included the cases without clear diagnosis such as unspecified anaemia. As a result, the diagnosis for D60-D64 (aplastic and other anaemia) included relatively more cases. For women before and after birth, unspecified common anaemia such as anaemia complicating pregnancy, childbirth and the puerperium (O99.0) may be included in this group.

Some researchers mentioned that hospital admissions for PND might be over-enumerated because hospital admission could occur for other medical reasons in the postnatal period [45, 46]. Furthermore, there was discussion over whether PND is a specific disorder distinct from major depressive disorder [47, 48] and maternal fatigue [49]. To minimise the potential overestimation of the rates, we only included the admissions with a ‘principal’ diagnosis of depressive disorders. On the other hand, the PND rates reported here might be under-estimated rather than over-estimated because hospital admissions would most likely reflect severe cases of PND [50] and majority of women experiencing PND were undetected and un-referred for mental health treatment by their obstetrical providers [51].

Temporal order between anaemia and depression could not be investigated by this study because majority of anaemia were diagnosed later than its occurrence. Majority of anaemia did not be found until women were admitted to hospital for giving birth or other reasons.

### Public health recommendations

This is the first population-based study to demonstrate a possible link between anaemia and depression in Australia, and emphasizes the importance and inter-relationship of both mental and physical health in the period before and after birth. Current clinical care practice guidelines recommend psychoeducation on the importance of iron during pregnancy and iron screening for women at risk of anaemia [52], as well as routine screening and monitoring of depressive symptoms [53]. Our findings highlight the important role of primary care providers in assessing for both anaemia and depressive symptomatology together, given the relationship between the two. Knowledge of the psychiatric implications of iron status in the postnatal period is critical given that approximately one-fifth of Australian women are already iron-deficient at the start of pregnancy [54], and PND can also be chronic for a subgroup of women [55].

## Conclusion

Anaemia was associated with postnatal depression for women. This finding highlight the important role of primary care providers in assessing for both anaemia and depressive symptomatology together, given the relationship between the two. Treating or preventing anaemia may help to prevent postnatal depression.

## Abbreviations

ABS: Australian Bureau of Statistics
ACPCP: Anaemia complicating pregnancy, childbirth and the puerperium O99.0
APDC: Admitted patients data collection
CHeReL: Centre for Health Record Linkage
CI: Confidence interval
HPA: Hypothalamic–pituitary–adrenal axis
MDC: Midwives data collection
NHMRC: The National Health and Medical Research Council
NSW: New South Wales
PDD: Postnatal depressive disorders
PND: Postnatal depression
SNOMED CT: Systematized Nomenclature of Medicine – Clinical Terms
